# Experiences of Norwegian Mothers Attending an Online Course of Therapeutic Writing Following the Unexpected Death of a Child

**DOI:** 10.3389/fpsyg.2021.809848

**Published:** 2022-01-27

**Authors:** Olga V. Lehmann, Robert A. Neimeyer, Jens Thimm, Aslak Hjeltnes, Reinekke Lengelle, Trine Giving Kalstad

**Affiliations:** ^1^NIEFT - The Norwegian Institute of Emotion Focused Therapy, Bergen, Norway; ^2^Portland Institute for Loss and Transition, Portland, OR, United States; ^3^Centre of Crisis Psychology, Faculty of Psychology, University of Bergen, Bergen, Norway; ^4^Department of Psychology, UiT The Arctic University of Norway, Tromsø, Norway; ^5^Department of Clinical Psychology, Faculty of Psychology, University of Bergen, Bergen, Norway; ^6^Centre for Interdisciplinary Studies, Faculty of Humanities & Social Sciences, Athabasca University, Athabasca, AB, Canada; ^7^The Norwegian SIDS and Stillbirth Society (Landsforeningen Uventet Barnedød - LUB), Oslo, Norway

**Keywords:** grief, therapeutic writing, stillbirth, unexpected death, online interventions, bereaved parents, motherhood, peer support

## Abstract

The unexpected death of a child is one of the most challenging losses as it fractures survivors’ sense of parenthood and other layers of identity. Given that not all the bereaved parents who have need for support respond well to available treatments and that many have little access to further intervention or follow-up over time, online interventions featuring therapeutic writing and peer support have strong potential. In this article we explore how a group of bereaved mothers experienced the process of participating in an online course in therapeutic writing for the integration of grief. Our research questions were: How do parents who have lost a child experience being part of an online course in therapeutic writing? What are the perceived benefits and challenges of writing in processing their grief? We followed an existential phenomenological approach and analyzed fieldwork notes (*n* = 13), qualitative data from the application and assessment surveys (*n* = 35; *n* = 21), excerpts from the journals of some participants (*n* = 3), and email correspondence with some participants (*n* = 5). We categorized the results in three meaning units: (1) where does my story begin? The “both and” of their silent chaos; (2) standing on the middle line: a pregnancy that does not end; (3) closures and openings: “careful optimism” and the need for community support. Participants experienced writing as an opportunity for self-exploration regarding their identities and their emotional world, as well as a means to develop and strengthen a bond with their children. They also experienced a sense of belonging, validation, and acceptance in the online group in a way that helped them make sense of their suffering. Online writing courses could be of benefit for bereaved parents who are grieving the unexpected death of a child, but do not replace other interventions such as psychotherapy. In addition to trauma and attachment informed models of grief, identity informed models with a developmental focus might enhance the impact of both low-threshold community interventions and more intensive clinical ones. Further studies and theoretical development in the area are needed, addressing dialogical notions such as the multivoicedness of the self.

## Introduction

The unexpected death of a child is one of the most challenging losses as it fractures survivors’ sense of parenthood and other layers of identity. Given that not all the bereaved parents who have need for support respond well to available treatments and that many have little access to further intervention or follow-up over time, online interventions featuring therapeutic writing and peer support have strong potential. In general, bereavement is one of the life transitions that make us the most vulnerable, as we touch upon the painful and often ineffable aspects of our human condition. Embracing the death of someone is a boundary experience, as it represents a rupture in the flow of our everyday lives, as well as in our imagined futures (Yalom, 1980; Zittoun, 2006; Lehmann et al., 2020). The death of someone close to us involves a rupture not just in the flow of our lives, but also in our sense of self, and therefore new narratives of identity must be crafted (Neimeyer, 2001; Lengelle, 2021), a process which often elicits resistance (Stroebe and Schut, 1999). When it comes to the unexpected death of a child, worldwide, all-cause mortality of infants was reported to be 4.8 per 1000 live births in 2012, which is one of the latest reports available (Müller-Nordhorn et al., 2020). In Norway, in average, there are 2.2 children under the age of five who die per 1,000 live births (UNICEF, n.d.). This means, for instance, that 314 unexpected deaths of children were reported in Norway in 2019, including 173 perinatal losses, as well as 131 deaths of children between the ages of 0–4 years from various causes (Norwegian Institute of Public Health, n.d.). Even if the average death of children in Norway is lower compared to other countries, there are several bereaved families who are in need of support in this country and whom unfortunately do not always receive the kind of support they need. Among other reasons, this is so because bereaved persons are often treated for depression instead of grief, and more tailored treatments for grief symptomatology itself are to be developed and offered (Hansen et al., 2020).

Although the emotional impact of the unexpected death of a child has been widely documented, there are few specific protocols for how to provide emotional support to parents in general and mothers in particular (Corno et al., 2020). If bereaved parents do not receive appropriate support and follow-up, they can experience fatigue, negative body-image, sick leave, unemployment, marital problems, and substance abuse, among other detrimental health and wellbeing outcomes (Burden et al., 2016; Dyregrov et al., 2020). Despite these known harmful effects, there are limited services to provide bereaved parents with the support they need. One of the challenges in providing effective intervention is the contradictory understanding of what such an experience of grief involves for the bereaved parents themselves. For instance, on the one hand existing theories suggest that this kind of grief can be harder than others because the death of children is unexpected, but bereaved parents can sometimes feel judged or misunderstood when told that their suffering should not be as it is, given the child did not live for a long time, and the bereaved therefore only related to the deceased for a short period (Kofod and Brinkmann, 2017; Brinkmann and Kofod, 2018). Bereaved parents often experience ambivalence as they try to find hope and meaning. Many parents feel a need to have protected or saved the life of the baby, while being confronted with the responsibility of taking care of themselves as well as taking care of their responsibilities and relationships with others as they grieve (Nuzum et al., 2018).

However, the nature and process of such ambivalence as it is experienced by bereaved parents is yet to be explored in depth. In recent decades, diverse theories of grief and bereavement have started to focus on process-oriented rather than stage-oriented models, drawing on idiographic and qualitative approaches to better understand processes such as grieving and mourning (Neimeyer, 2001). Paying attention to individual differences and more detailed accounts from study participants could enrich the theoretical understanding of trauma and loss, as well as improving the effectiveness of interventions (Bonanno, 2004). In addition, there is a need for qualitative analysis of interventions for the bereaved, with a cultural and community focus (Human et al., 2014). Amongst them, therapeutic writing groups offer diverse possibilities. Writing practices are effective tools to find meaning after experiencing boundary experiences, such as the death of a loved one (Meijers and Lengelle, 2012). The general therapeutic effects of writing have been well documented (e.g., Pennebaker, 1999; Ullrich and Lutgendorf, 2002), and specific evidence about its effects as part of interventions to process grief and bereavement has also been widely documented (e.g., Neimeyer, 2016; Den Elzen, 2018; Lengelle, 2021).

There is little research about the experiences and effects of therapeutic writing among bereaved parents whose children have died unexpectedly, even if some expressive writing practices as part of larger workshops have proven to be of benefit (Dyregrov and Gjestad, 2015; Neimeyer and Young-Eisendrath, 2015). In addition, even when receiving this or other kinds of mental health support, bereaved parents are among the groups whose grief symptoms are the hardest to treat, especially if they experience symptoms of prolonged grief, i.e., persistent and intense grief reactions (Shear et al., 2005; Johnsen et al., 2012; Dyregrov and Gjestad, 2015). Among other factors, the sudden death of someone as well as the lack of social support to integrate the grief are risk factors for developing prolonged grief (Burke and Neimeyer, 2013). One of the possible reasons for these hardships in treatment outcomes could be the need for an expanded understanding of the complexity of the emotional processes that constitute grief as a phenomenon (Brinkmann and Kofod, 2018). Therefore, there is a great need to expand knowledge of the emotional challenges of facing the unexpected loss of a child – such as is the case of perinatal loss, illness, or accident – as well as to innovate interventions that respond to these emotional needs. In this article we explore the following questions aimed at expanding the current psychological knowledge in this area of grief: *How do parents whose children have died unexpectedly experience being part of an online course in therapeutic writing in Norway? What are the perceived benefits and challenges of writing in processing the grief among course participants?*

### Therapeutic Writing for the Integration of Grief

Writing courses are low-threshold interventions which could have great impact in the promotion of mental health and well-being because they hold space for the uniqueness of human experiences to be expressed, crafting a sense of reciprocity (Lehmann and Brinkmann, 2019a). However, not all writing practices are as beneficial, and some forms of “free” writing might lead to rumination among other impediments to meaning-making and affective processing (Nolen-Hoeksema, 2001; Pennebaker and Smyth, 2016). Thus, it is important to combine both practices of free-writing and directed-writing, to facilitate insights among participants, and to target the individual needs of participants in relation to their own vulnerabilities (Furnes and Dysvik, 2010). When it comes to grief, mourning and bereavement, writing courses address the importance of telling one’s stories of grief and finding an audience for such stories, which is a crucial part of most therapeutic processes (Neimeyer, 2001). In doing so, we write storylines about our lived experiences from different parts of the self (Lengelle, 2021). One of the goals of therapeutic writing around grief is to bring these diverse parts of the self – referred to in Dialogical Self Theory (Hermans, 2001) as I-positions – into dialogue and to turn default narratives into more live-giving stories (Lengelle, 2020). More qualitative knowledge is needed about the sorts of dialogues that this multi-voiced self can engage in the process of finding meaning in life through and despite grief (Neimeyer, 2011).

Writing courses, for instance, have been widely used as part of individual and group interventions to support bereaved people as they engage in the process of both orienting themselves to process the grief and reconstructing their lives (Neimeyer, 2016). There are different ways to structure a course of therapeutic writing for bereaved people. For example, participants might be asked to describe their psychological reactions in relation to the grief, such as their innermost feelings around it, as well as what they can learn or have learnt from such experience (Neimeyer and Konopka, 2018; Lengelle, 2020). Other directive questions could involve inviting participants to write a letter of advice to other parents in a similar situation (Dyregrov and Gjestad, 2015). In addition, writing can recruit metaphorical resources, as in the case of poetry, to transcend the limits of literal language to voice the heart of grief (Neimeyer, 2001; Lengelle, 2021). Developing group-based writing courses likewise is important because it can offer an opportunity for emotional processing and meaning-making, as well as developing a network of peer support during and after the course. Given that bereavement, mourning, and grieving can be challenging for the individual, community endeavors can be a great resource in both processing the grief and in reconstructing life beyond it. Gaining knowledge about this process can make interventions more accessible, especially if online implementation proves to be as effective as the face-to-face alternatives.

### Online Interventions in Mental Health and Grief Therapy

When it comes to interventions in mental health, there is growing interest in the potential of internet-based interventions to reduce the gap between the people who need mental health support, and the people who receive it (Berthoud et al., 2021). Even at this early point in research on the efficacy of such therapeutic offerings, online interventions as low threshold interventions to treat diverse forms of grief have proven to be effective (Eisma et al., 2015; Wagner et al., 2020; Treml et al., 2021). As one means of helping participants integrate the grief and build community, such online interventions have obvious advantages in feasibility, cost effectiveness, and accessibility, especially for those bereaved parents who do not live in major urban areas. For example, online interventions have become an effective tool to reduce the loneliness experienced by many bereaved groups during the COVID19 pandemic, enabling the participants in these interventions to build relationships that support them in their grief (Berthoud et al., 2021).

In the spring of 2020, the first author received funding from Stiftelsen DAM in Norway, in collaboration with the Norwegian SIDS and Stillbirth Society (Landsforeningen uventet barnedød - LUB) to develop and run a pilot online course in therapeutic writing to support bereaved parents who were lacking support given the social isolation that arose from the COVID-19 outbreak. Given the substantial interest bereaved parents expressed in the online course, the project was extended through research funding from both LUB research fund and the DAM Foundation. Two further editions of the course were run, and here we document the experiences and challenges of participants who attended to these two latter courses.

## Materials and Methods

### Methodology

We followed principles of existential phenomenological research in both psychology (Osborne, 1990; Giorgi, 2012; Churchill, 2022) and anthropology (Jackson, 2012; Usher and Jackson, 2014; Jackson and Piette, 2015). From this standpoint, we explored the lifeworlds of each of our study participants aiming at understanding in depth their intentions, desires, and life projects, and how these are influenced by historical and sociocultural conditions (Jackson and Piette, 2015). Life’s unfolding in consonance or dissonance with our intentions and desires, sooner or later, puts all of us human beings in touch with existential givens such as uncertainty, death, loneliness (Yalom, 1980; Cooper, 2017). Consciously or not, we take different paths as we make sense of these experiences, and when integrated in terms of virtues, these pathways can transform our attitudes and choices (Lehmann and Brinkmann, 2019b). Our aim has been to explore with as much empathy and compassion as possible, how parents experience their grief and mourning, as well as the possibilities and impossibilities they face as they try to find a meaning in their grief and make meaningful their everyday lives despite them.

### Study Participants

Our study participants were recruited through an online application survey the organization LUB shared with their members and followers in social media. Most of the participants were already on a waiting list, given their interest in attending a pilot writing course that the first author of this article facilitated during the summer of 2020. The inclusion criteria for this study involved having lost one’s child at least 2–3 months prior as well as readiness both to share one’s writings and lived-experiences with others in the class, and to listen to the writings and lived-experiences of others, which requires tolerance of a wide spectrum of feelings and emotions. All those interested in participating from the study filled up the Norwegian translation of the ICG- Inventory of Complicated Grief, which has been used in other published studies and also later on validated to Norwegian population (Dyregrov et al., 2003; Thimm et al., 2019). Both participants with symptoms of complicated grief and those who did not score as high in the inventory were admitted in the study, may they comply with the other inclusion criteria. Exclusion criteria were, for instance, active symptoms of psychosis or suicidality, death of a child due to suicide, death of a child over 10 years ago, or unavailability to connect to the online sessions according to the research schedules. Out of the 45 people who applied were admitted into the research project after undergoing screening interviews by videocall. Using the “Research randomizer” website (Research Randomizer, n.d.), the participants were randomized into two groups of 17 (group A) and 18 (group B) participants each. In the study 28 participants completed the 8-week program, 15 participants from group A, and 13 from group B. Among the reported causes for withdrawal were responsibility for sleep time of other children, as well as illness, or a need to prioritize other kinds of mental health support. In Table 1, we provide the sociodemographic information of the participants.

**TABLE 1:** Sociodemographic information of the study participants.

Heading	Description
Total participants	35
Gender	Female
Age	n/a, 6% (*n* = *2*)*;* 20–25, 6% (*n* = *2*)*;* 26–30, 17% (*n* = *6*)*;* 31–35, 31% (*n* = *11*); 41–45, 14% (*n* = *5*); over 45 years 6% (*n* = *2*).
Year of death of children	2016 or prior, 24%; 2017, 9%; 2018, 23%; 2019, 9%; and 2020, 32%.
Cause of death of children	Stillbirth 70%; illness 19%; accident 3%, and other cause 8%.

*1 participant had lost 3 children and 2 participants had lost 2 children and therefore all children were included in the data about year and cause of death.*

### Developing an 8-Week Online Course of Therapeutic Writing to Support Bereaved Parents

The 8-week online course was developed and facilitated by Dr. Lehmann under supervision of Dr. Neimeyer and Dr. Lengelle. The structure and contents of the course also benefited from user insights from the 14-week pilot of the course that preceded this study. For instance, other than providing feedback about the writing practices, these users identified anger and shame as the most difficult emotions in their experiences of grief and explicitly suggested that these topics be covered both in terms of psychoeducation and writing practices.

In addition to the literature on constructivist meaning making and therapeutic writing (Neimeyer, 2001, 2012), this course integrated knowledge from a variety of traditions: mindfulness and compassion-based approaches to psychology (Chozen Bays, 2009; Neff, 2016; Weiss, 2018), emotion focused therapy (Greenberg, 2011), as well as meaning-oriented psychotherapies based on the Dialogical Self Theory (Hermans et al., 1992) and Logotherapy and existential analysis (Frankl, 1969). In practice, these theories were introduced to the attendants in the forms of virtues such as hope, love, self-exploration, compassion, and self-compassion. Principles and models that informed the work theoretically included the dual process model of coping with bereavement (Stroebe and Schut, 1999) and the continuing bond model of grief (Klass et al., 1996). The integration of these perspectives was then tailored to be low-threshold community intervention; that is, our online course on therapeutic writing was neither designed to be a group therapy process, nor a clinical intervention.

Each session lasted for about 3 h, including two short breaks. We used the software Zoom as a teaching platform. In summary, the course structure consisted of a 5-min mindful transition, followed by a journaling check-in during which participants noted their feelings before starting the course. Then we usually had a brief plenary discussion about the class theme, followed by individual work with the writing tasks. After participants had worked on their writings, they were randomly assigned to a breakout room to share their insights with others in a small group. In these smaller groups, they had time to listen to one another’s texts, as well as reflect on how they experienced the writing practices. Then we returned to plenary sessions where the study participants had opportunities to give feedback to the course facilitator and share their experiences about the writing practices. Participants were urged not to read their texts out loud unless they felt ready to do so. Rather, they were encouraged to share how they experienced writing itself: their resistances, the feelings that arose, their insights, and so on. In what follows, we summarize the main contents of the course.

During weeks 1 and 2 we focused on psychoeducation around grief and therapeutic writing. We also established group guidelines such as confidentiality, respect for their own emotional rhythms and finding a pace for words and for silence. We also established mindful listening as a practice by which participants could honor and hold space for the texts and reflections of one another without interrupting to speak of their own experiences or jump into problem-solving. We used writing practices such as writing an acrostic poem with each of the letters of the alphabet that form the name of their children. Another of the practices was an adaptation of proprioceptive writing (Metcalf and Simon, 2002). In this, participants listened to a piano piece by Ludovico Einaudi, then wrote the story it inspired about their grief. To enhance their writing, they were encouraged to use the proprioceptive prompt to deepen the meaning of some of the words they used: “What do I mean by “X”? By “X” I mean.”.

During weeks 3–5 we focused more explicitly on meaning-making and dialogical psychology, using the metaphor of the mind being a theater with different characters such as various versions of ourselves or others, or diverse emotional tonalities. For instance, we read the poem “The Guest House” by Rumi (Jalāl al-Dīn and Barks, 1996) after which participants were asked to reflect upon diverse guests in the inner house of their minds, such as parts of themselves that have become more silent as they grieved. In addition, we worked on emotions such as anger or shame with a focus on compassion and self-compassion. Here we did a modified version of the practice “How would you treat a friend?” (Neff and Germer, 2018), where they wrote letters showing compassion to another course participant. Then they reflected upon whether they are as compassionate with themselves in their grief as with others, and how they might treat themselves with more friendliness and loving-kindness. We also did a modified version of the exercise, “Introducing the loved one” (Hedtke, 2012), in which the participants wrote in the third person about themselves and their relationship to the children they have lost.

During weeks 6–8 we focused more explicitly on existential meaning and purpose. Some of the practices we included involved the technique “Life chapters” which has been used in meaning focused grief therapy (Neimeyer, 2011). In this writing practice, participants were invited to imagine they were 85 years old and writing their autobiography. What would be the table of contents of such a book? Other practices involved learning first about the English acrostic “VERB,” which stands for Victimhood, Entitlement, Rescue and Blame mindset, representing the pitfalls in narratives that increase our suffering and disempower us (Baker and Stauth, 2003). In therapeutic writing, the model of transformation through writing maintains that boundary experiences, such as the death of a loved one, prompt individuals to create first narratives often characterized by elements of VERB, while the aim is to develop “second stories” i.e., narratives where a life-giving perspective of resilience and understanding takes shape (Lengelle, 2020). In the final session, we wrote a group poem with the prompt “a door opens,” in which each of the participants contributed a stanza.

### Data Collection

The main source of data in this article are the fieldnotes that the first author wrote as she facilitated the two rounds of the 8-week course in therapeutic writing. In total 13 journal entries out of the 16 weeks of teaching were included (journal entries were missing/illegible for week 7 in course A and weeks 1 and 3 in course B). These fieldnotes summarize the first author’s observations and reflections about what participants shared in the sessions. In existential phenomenological anthropology and ethnomethodology one of the core functions of fieldwork is to report and give voice to the world from the point of view of study participants (Kawulich, 2005; Jackson, 2012). This requires that the researcher record observations with enough openness and inclusivity, as well as the sensitivity to craft relationships of trust with the participants who are opening about the most intimate and at times secret aspects of their lives (Jackson, 2012). Therefore, in ethnomethodology the researcher has a dual focus: to describe what the participants give voice to, and to interpret and shed light on what they cannot yet put into words (Jackson, 2012; Jackson and Piette, 2015).

To foster the validity of the fieldnotes, we triangulated the data with other information sources, such as the application (*n* = 35) and assessment (*n* = 21) surveys, excerpts from the journals of some participants (*n* = 3), and email correspondence between the participants and the course facilitator/main researcher (*n* = 5). In a nutshell, the application survey included questions about participants’ expectations about the course, as well as sociodemographic information. The assessment survey addressed questions such as which practices the participants experienced as most meaningful, what they felt had worked well, and how such a low-threshold service can be improved. In addition, three participants made excerpts of their journals available: two of them gave stories they wrote about their grief, and one of them offered a table summarizing her weekly check-in and check-out journaling in class.

### Data Analysis

As suggested in the procedures of data analysis of phenomenological research (Fischer and Wertz, 1979; Osborne, 1990), the main researcher transcribed the fieldnotes and categorized them into three different groups: (a) descriptions of the experiences of the participants, including their own words as shared at class; (b) a summary of the transcriptions; (c) preliminary themes condensing the main meaning-making processes of such experiences; and (d) feedback from the participants about the course structure and online setting. Then, she read these files several times to get a feeling of the data and created individual case synopses for each of the two writing courses, week by week, to have a longitudinal overview of both the contents and the processes to which the data referred. In Table 2, an example of the process of structuring the fieldnotes is provided. Finally, we created general condensed summaries of meaning, integrating the separate analyses of both courses, which were organized in the following themes suggested by the wording and metaphors invoked by participants: (a) where does my story begin? The “both and” of silent chaos; (b) standing on the middle line: a pregnancy that does not end; (c) closures and openings: “Careful optimism” and the need for community support. The contents of the three excerpts of the journals from the participants, as well as the qualitative answers to the application and assessment surveys, were integrated into these themes to facilitate the triangulation of the data.

**TABLE 2:** Example of meaning condensation and structure of the fieldnotes.

Group A		

Fieldnote transcription week 5	Summary	Analytical remark
For some participants it was difficult to write in the 3rd person, as if a friend were writing to them. Their challenge was related to the idea of a friend, rather than to the third person instruction itself. For many it was actually easier to write in the third person than in the first, as it gave them feel more expansive opportunities to write and find flow. “It is easier but vulnerable to come closer to grief this way” one said. Most highlight that to think about “instants” where one forgets to grief, or when one feels something, was helpful. Maybe give more time to work on such instants? Focusing on expectations, they appreciate my validation that it is okay just to survive some days, to focus on survival some days.	Third person practices as effective ways craft distance enough for them to get in contact with their experiences: Detaching and externalizing. Appreciation of validation about wherever they are at in their grief. Appreciation of the instants where one either forgets or reconciles with the grief.	Meta-perspective as a dialogical endeavor. Appreciation of the instant, as placing grief into context of their wider lives. Confirming need for validation

**Group B**		

**Fieldnote transcription week 5**	**Summary**	**Analytical remark**

Practice of “she who grieves” leads to more flow, an acknowledgment that they want to understand as an observer, and they can understand more of themselves. It is also a confirmation of the loneliness they feel and at the same time a dialogue, which was the theoretical insight I wanted to convey. Distance helps to write, easier to write in the third person for some, and friendlier and less self-critical than other texts. But for a couple of them it felt less personal, like not so attached to the child. Another one had a feeling that they needed to show others and find words so they can feel understood. Easier to feel empathy and compassion for themselves. A way to acknowledge that they care about the child, and at the same time accept what has happened, to be in contact with the reality of the loss. For some it was strange to formulate in the third person. For others it is harder to do it in the first person given the vulnerability. Some participants say that the “think as if you were a friend” guideline was ambivalent because then they are drawn to a specific person, even if we meant it as a metaphor.	Restoring the dialogical quality of the mind, by detaching from the first person. For most of them, this distance gives room to more emotional immersion and facilitates writing, but for a couple of them, it creates disconnection from the child and resistance.	Not all of me grieves: meta-positioning. Theoretically this speaks again about resistance and identity

### Bracketing the Lifeworld of the Main Researcher

Existential-phenomenological research highlights the presence of the researchers, their own lifeworlds and knowledge as a potential resource for the research. It is the rigorous self-reflection of the researchers, the awareness of their biases, that can contribute to a deep understanding and interpretation of the lived experiences of study participants (Osborne, 1990). Hence, we provide a reflexivity statement from the facilitator of the courses and main researcher in the project:

*It is inevitable for me to look at the symptoms of grief from the perspective of my life course. This is so, given my personal experiences with grief and loss, my background in clinical and developmental psychology, and my insights following a group of bereaved mothers for 14 weeks prior to starting up this research project. I have not experienced the unexpected death of a child, but I remember with mixed emotions the two times I feared being pregnant and my ex-boyfriend said that had I been pregnant he would not want that child—once because it was too early in our dating history, and by the time we broke up, because he did not see a future with me. I did not lose a child as I was not pregnant after all, but I lost the possibility of future motherhood with him, and becoming single, suddenly the image of any other form of involuntary childlessness became daunting to me.*

*As a young adult who has not had a chance to have children yet, I have explored the crossroads of motherhood for my identity and that of other women in collaborative autoethnographic accounts* (*Lehmann et al., 2020*). *Facilitating three courses on therapeutic writing, first the pilot and then the two courses included in this study, the question of motherhood kept beckoning to me: Will I remain “only” an aunt or a godmother? Do I, indeed have a choice? What if I am sterile, or my future partner is, or what if I, like my study participants, experience the unexpected death of a child? The study participants wonder if I have been through the same experience they have, a couple of them asked and I limited myself to emphasizing that whether I have experienced it is not as relevant as is my purpose to learn from them, to accompany them in their grief through writing. I have experienced many other kinds of grief and trauma in my life and still, beyond my genuine efforts to be empathic and compassionate, I do, at times feel an outsider to the intensity of their pain. What the study participants and I have in common is that we are all making sense of what has and has not occurred in our life trajectories. In developmental psychology there is the notion of a shadow trajectory* (*Bastos, 2017*), *which emphasizes how the intensions and desires that have not been realized in our lives affect our sense of identity. I attended to the quest of my study participants to find meaning in their grief, which transformed their sense of being in the world, and did so with as much empathy and compassion as possible. I shared with them openly my theoretical perspectives, so that, as we navigated the 8-week program we co-constructed meanings together. Our dialogues in the plenary sessions week by week were then fueled by their vulnerable recognition that while grieving they felt lost as human beings, that they did not feel like themselves and wanted to find their way not only through grief, but also through life.*

*As a teacher, I invited them to navigate the waves of grief, uncertainty, and impermanence through writing. As experts in their pain and in their own life, they invited the researcher in me to suspend my assumptions and descend to the underground, the Hades, of their experience. At times they even asked me to stop inviting them into the “light” even if they appreciated the sweet intention of bringing them hope. They needed me to stand in the darkness with them, and this research project became in large part an effort to expand the current knowledge we have about what makes this kind of grief one of the most challenging to treat.*

## Results

### Where Does My Story Begin? the “Both and” of Their Silent Chaos

According to information from the application surveys, learning techniques in therapeutic writing was only one of the motivations for study participants to enroll the course. Beyond writing they saw this course as an opportunity to receive psychoeducation around grief in the hope of finding words to help accept the death and cope better with their everyday lives. For example, to the question of what kinds of topics they believed would be important for them to integrate their grief during the writing course, one participant wrote “*Who am I/who will I become without my child.*” Similarly, but emphasizing more the context of her search for meaning, another one specified: “*I want to describe and try to find words for how it is to lose a child to stillbirth at the age of 40. An age that is not necessarily the best one if one wants to become pregnant naturally.*” At the first plenary session, when being told that during this course on therapeutic writing they would focus on at least three stories: the story they needed to tell, the story they wanted to tell, and the story that others could learn from, many participants said that they “*want to feel like themselves again*” or “*find themselves again*,” and one study participant asked out loud: “*But, where does my story begin?*” which inspired the title of this portion of the results as it was central to participants’ experience.

Previous qualitative studies have also reported that bereaved people can feel lost in their sense of identity, even if more information about the processes regarding such identity loss and its reconstruction are needed (Gillies et al., 2013; Lengelle, 2021). With this as a background of identity loss and a painful yearning to find the right words to tell a story, it is no wonder that at the beginning of the course, resistance and chaos was a common experience among the study participants. For example, at the first plenary sessions in Zoom, participants referred to a fear of entering the dark room of their grief, losing control of their chaotic emotions, as they would write. The paradox is that one of the main motivations to enroll the course, according to the answers to the application survey, was to find words, a wish to better understand grief, while also having tools to better communicate about their needs and wants with others in different contexts. Such a search to find words, a silence often described as chaotic, was experienced as the possibility to honor the simultaneous coexistence of feelings and emotions, as well as unresolved and seemingly meaningless episodes of their life stories. Most of them named struggles in coping with an experience they continued to find traumatic, which affected their couple relationships, their work, their interactions with the social and health care systems, as well as with friends and other family members. Indeed, the nature of intense emotional processes such as grief can transcend the capacity of language (Jackson, 2004; Lehmann, 2018a) and give people an experience of being frozen in time (Van der Kolk, 2015). Validating their struggle to find words, I suggested that they honor the silence that was part of their grief, which released them slowly from the performance anxiety that they voiced during the first weeks of the course. At this point, most of them acknowledged their need to process not only their sadness, but also other emotions such as the anger, guilt, and shame related to it. For example, one study participant wrote the following directed to her stillborn child:

*I’m really mad at you. That you did not make it this fucking time*, *that you did not grow as you should when it seemed like everyone else was growing.*

*Why did you not do that? Young bastard.*

*I hate you!*

*Shame.*

*The guilt for the anger and rage I now feel.*

*It was never your fault.*

*The fear of how doomed I would be if anyone read this.*

*Arg, arg, arg, arg, arg.*

*Damn hope*

The anger, guilt and shame that blend in this sharing evoke the challenges in finding hope in the grief, as well as how difficult it can be to make sense of unmet expectations about raising a healthy child. Finding benefits and possibilities of growth after the death of a loved one is actually very difficult for bereaved people whose grief contradicts the expected cycle of life (Gillies and Neimeyer, 2006). As this example illustrates, writing their way into their emotional world, no matter how confusing or chaotic it can feel at times, was not necessarily about finding meaning, not yet. It was about acknowledging their human condition, as they found themselves in the dark labyrinth of traumatic loss, searching for meaning. For instance, most participants in both groups referred to an openness to embrace most of the virtues introduced at class, but not gratitude. In the third week out of the eight that the course lasted, we read Rumi’s poem The Guest House to introduce a metaphor of the different dimensions of their identity (e.g., mother, sister, painter, and dancer), as guests of their inner house. Rumi’s (Jalāl al-Dīn and Barks, 1996, p. 109) closing verses in the poem stand:

“*Be grateful for whoever comes*

*because each has been sent*

*as a guide from beyond.”*

Participants from both online courses had similar reactions to the poem, which opened a Dialogue about the possibilities and impossibilities of finding meaning after the death of their children. They described in the plenary sessions feeling pressured by family, friends, their health care practitioners or at work, to be grateful about being able to at least experience what a pregnancy is like. However, gratitude is perhaps the virtue that made least sense to them, given the absurdity and unfairness of their tragedy. It takes time, sometimes years, to find benefits or new meanings after a traumatic loss and to integrate it into one’s sense of self (Gillies and Neimeyer, 2006). Therefore, what became most valuable for the study participants during the first weeks of the online course was the space to explore and listen to their own emotional rhythm through writing. It was crucial that they allowed themselves to express what they felt and not what they thought others expected them to feel, while also giving themselves time to find words and to recognize that sometimes expressing confusion and overwhelm is enough. Giving themselves permission to write, to stay silent, and to honor that it takes time to find words or to find meaning in the tragedy they are recovering from was what they could feel gratitude for. As words found their way into oral conversations and journal entries, the study participants discovered that not all of their feelings, thoughts of behaviors were to be treated or healed. Rather than an “either or” process, grief became a “both and” process, where the acknowledgment of their human condition became an existential need met by the peer support and the facilitation of the writing and sharing practices. “*I thought I was going bananas and now in this course I find relief in learning that I am a human who grieves*” said once a participant at class, and many resonated with her.

As they engaged more and more in the writing practices and oral discussions, they connected with a sense of hope for finding meaning in the grief while discovering possibilities to nurture a bond with their child. Experiencing the possibility of a continuing bond with their child appealed to them, as affirmed in other grief studies, functioning as a form of meaning-making and motivation (Neimeyer et al., 2006; Paxton, 2018). For instance, according to the assessment survey, for the great majority of participants, practices such as writing an acrostic with the letters of the names of their children were amongst the most meaningful activities for the participants [81% (*n* = 17 out of 21)].

### Standing on the Middle Line: A Pregnancy That Does Not End

As the weeks passed and we approached the midpoint of the course, the participants’ sense of resistance and ambivalence prevailed. They acknowledged further their need to be in contact with the reality of grief, to accurately describe the story of their raw pain, as well as their contrasting urge to avoid the pain associated with diving into the reality of the death of a child. In Table 3 we provide the excerpts of the journaling check in and check out of a study participant during the weeks 4 and 5 of the course.

**TABLE 3:** Journaling summary from the check-in and check-out prompts.

Week	Check in: I feel… about		Check out: what I learnt today about…		
	Grief	The course	Grief	Writing	Myself
4	Afraid of, angry about. Neither ready nor at peace with it.	Reluctant, hesitant. No enthusiasm, no safety.	There’s a lot of anger in my grief, perhaps more than I would like to recognize.	Writing helps me to look at myself. Therefore, I need to write. I have to write!	No matter which situation I am in, I will always fear that others would think I am weird, silly or irritating, and I think too highly of myself.
5	Afraid of grief, Lonely.	Tired, Lack of interest.	Grief comes in waves. Don’t be afraid, I will get to rest in a little while.	To write in 3rd person is useful. This distance gives me another entrance point toward myself.	I have something to contribute, and that is meaningful for others. When I am honest about my shame, the others feel less alone in theirs.

As these excerpts illustrate, one of the main insights that participants gained through the writing practices was the awareness of the fleeting nature of feelings and emotions, and how powerful their self-criticism could be. In the plenary discussions, they would often recall that it felt safer to speak about their grief as a result of their sense that they had the group and facilitator’s non-judgmental support. There were at least three different layers of validation that the course work provided them. First, psychoeducation on the dual process model of grief (Stroebe and Schut, 1999) made them feel seen, becoming a great tool for them to understand that this feeling of resistance was part of grieving itself, and not something that made them “crazy.” Second, they experienced such validation of their ambivalence as a relief to the shame that they felt so strongly and seldom admitted or spoke about outside this online course. As an emotional process, shame is the feeling of being wrong as a person, a failure or less than others (Greenberg and Iwakabe, 2011), and those feelings at times come from unmet expectations or perceived societal pressure to feel or be something one does not in a particular situation. Third, psychoeducation about shame and the validation that given the tragic quality of the death if their children, it was no wonder that they might feel like a failure or that something was wrong with them, helped them to also understand their experience better. However, the perceived effects of psychoeducation and validation did not end with understanding. They also inhabited the ambivalence and tragedy of their experience with kindness and empowerment, which are elements of compassion and self-compassion (Neff, 2016; Lehmann, 2018b).

In particular, the focus on virtues such as love, compassion and self-compassion made them aware of their inner critic, emphasizing that: (1) it was easier to be compassionate with others in the course than with themselves; (2) the inner-critic was part of them before grief; (3) putting this into words helped them to commit to the practice of virtues such as loving-kindness or self-compassion. The following excerpt of a story written by one of the course participants gives an account of the importance of integrating grief in one’s everyday life. Magda Hognestad, who has explicitly requested us to use her real name given that she has published the full story in her social media and the social media of SIDS already, said that:

*“It is said that it takes 7 years for all the cells in the body to be replaced. If this is so, all of the cells in my body since the time Nikolai died have been renewed. This is something, you might think. And it is something indeed. I could as well say that has not been long, neither in my body nor in my soul. Pain, guilt, the exhausting grief has been unbearable. I did not understand how I was supposed to put myself together and then call this 1 week, 3 weeks, 2 months, 1 years, 3 years since he died. Someone once told me, some months after the 17th of February 2014, that it was a shame that I, a person who was so sensitive and cried so easily, had to experience this. Oh my God! If only this person would know how much strength, inner safety, care, and sensitivity is needed to survive a trauma like this, then I would have never been told that.*

*To survive the loss of one’s child is to constantly feel like two versions of yourself and of your life: the outer life that you eventually manage to sign up for again, and the inner life. After the shock subsides, you manage to develop a bunch of survival strategies, and this is what the inner life is about.”*

Some grief interventions focus on exposure to the painful memories of the death, such as narrative reconstructions of the traumatic experiences (Hansen et al., 2020). Others follow a more relational and dialogical approach (Neimeyer, 2011; Lengelle, 2021). In the writing course we covered both, but we devoted more time to compassion, validation of the participants emotions and focus on discovering sparks of beauty during the darkness of their grief (Neimeyer et al., 2018). As part of the process of developing self-compassion and compassion for other course members, the study participants and the course facilitator identified one important aspect of the deep sense of ambiguity that prevailed in them. Compared to other experiences of grief where the deceased person is older in age, the unexpected death of a child—whether to stillbirth, illness, SIDS, or accident—contradicts the expected course of life. As some participants further described in our plenary discussions, it is easier to anticipate the death of a person the older she gets, but harder to logically understand that someone can die soon before being born, at birth, or soon after birth (LaRocque and Burk, 2020). In trying to accept the illogical reality of the death of their children, many of them then experienced not only intense shame, but also anger. The tragedy of this ambivalence is that it leads to existential questions that they shared aloud in class such as: Do I have the right to grieve? What is the threshold between life and death? And, for those bereaved parents of a stillborn child: Was my son/daughter alive? Am I still a mother now that my child is dead? These questions evoked in them a feeling that their pregnancy did not seem to end; the nostalgia because a part of them is waiting for something that was supposed to occur and did not occur: the birth and continuation of the life of their child.

Even though most, but not all of the children whose parents were grieving during the writing course died to stillbirth, participants recognized a sense of belonging that facilitated meaning-making and emotional processing. Writing and sharing aloud their shame, as well as voicing these questions, crafted a sense of belonging and acceptance, these being, according to emotion focused therapy, among the core human needs that shame sheds light upon (Greenberg and Iwakabe, 2011). The key catalyst of this sense of belonging and acceptance for most participants was not feeling alone in their experience. Writing in a group setting conveyed a sense of belonging where they experienced reciprocity, giving, and support. In an earlier qualitative study on the effects of writing courses it has been found that self-exploration, otherness and togetherness are the key elements for promoting mental health and wellbeing (Lehmann and Brinkmann, 2019a). In keeping with those findings, our online courses enabled self-exploration, helping the study participants get in touch with their emotions associated with grief and find meaning and motivation to cope. In addition, they embraced a relational movement toward otherness, feeling motivated to support others in their grief throughout their stories, as well as with their mindful listening. They also described a sense of togetherness and community which they crafted across the 8 weeks, this being one of the most recurrent aspects for which they expressed gratitude in our evaluation survey. For instance, one participant wrote in the evaluation survey at the end of the course that: *“I liked how writing gave me insight and teachings about what is happening within. In addition, I liked especially the togetherness and community that I felt with the other participants.”* In addition, the attendees of both writing courses decided to establish self-organized online groups to keep in touch, to share their future writing, as well as to provide further emotional support to one another. This illustrates the power of togetherness and peer support and the necessity of multifaceted strategies in addition to clinical interventions to meet the different needs of the bereaved parents.

### Closures and Openings: “Careful Optimism” and the Need for Community Support

By the last session of the online course, our participants described in plenary a “*good tiredness*,” because it takes a lot of emotional energy to be part of such a course on therapeutic writing, but it also pays to have found more words and understanding about their grief and about themselves. For instance, a participant wrote this in the evaluation survey:

*“I especially liked that I got to know myself better as a person. That I have different characters, like a theater, and then I got to know myself better and understood better who I am. So, now that this is in place, I have a better starting point to also understand grief itself and work on it through different practices. I think this course managed to “divide” grief in suitable pieces, so that it became clearer and more feasible for me to relate to it. Some of these pieces are of course tougher than others.”*

According to this study participant, there is a sense of mastery over grief that arises from exploring the self through dialogical lenses (Hermans, 2001). Throughout diverse writing practices, the course attendees positioned themselves not only as mothers, but also as authors and storytellers, friends, partners, peers, as well as other dimensions of themselves related to hobbies and career. One of the main techniques used to restore the dialogical quality of the mind was introducing a character such as a friend, their child, another classmate, for them to engage with curiosity instead of judging their feelings and emotions, which also resulted in a better perception of flow in writing. Expanding the understanding of their identity helped them identify areas to which they had given either more or less attention in their lives, both before the grief or now in adapting to it. Given that the sense of identity of bereaved parents is often shattered after the death of a child, external symbols—such photographs, or in our study, writings—can help parents restore a bond with their children and with it, a part of their identities as well (Riches and Dawson, 1998).

However, this restoration of their sense of identity as parents is to be understood as an ongoing process that entails attunement with different difficult emotions that arise as part of bereavement, such as anger or shame. For instance, during the closing circle of the therapeutic writing course, one participant from the group B said she experienced “*careful optimism*” toward her life and her future, which summarizes the overall perceptions of participants in both groups. As the writing course came to an end, they expressed expanded understanding of grief and their lives. Rather than feeling “healed” or “over” or done with the grief, they got better acquainted with the nature of grief and its underlying waves of emotions. When comparing what she wrote in each check-in and check-out journaling sessions we had during of the 8 classes, still another participant noticed a transition from “*empty into something… something else that changed within.*” At times it was from emptiness to hope, or emptiness to anger, or emptiness to sadness. In Table 4 we can see an example of such transitions more explicitly, as one participant noted in her journal:

**TABLE 4:** Journaling summary from the check-in and check-out prompts.

Week	Check in: I feel… about		Check out: what I learnt today about…		
	Grief	The course	Grief	Writing	Myself
7	Angry, without hope. Neither accepting nor at peace with.	Tired and uncertain.	Grief will trump reason for a long time, and that is ok.	I should write more, not be so afraid of it, remember it feels good.	What I have learnt and understood is of value for others. I should keep sharing, even if I feel silly.

Other than anger or shame, the end of the online writing course could be summarized as in a sense of “careful optimism” because the suffering our study participants experienced did not come to an end. Therefore, as shared in the table above, participants who found value in therapeutic writing needed to intentionally remind themselves of its benefits and walk themselves through the fear, anger, guilt, shame, and other emotions that might interfere with the process. It is common to experience mixed feelings that influence decision-making, and to attempt to avoid memories of the baby (Burden et al., 2016). One of the ideas that resonated the most with the study participants was that it was possible for them, given that their identity was a theater with diverse characters, was to experience more than one feeling at once. Instead of either-or polarities such as “if I grieve I cannot/must not feel happiness,” it became possible for them to see that they could have one or several instants of happiness AND still grieve, still love, and honor the child they had lost. This was especially helpful for those attendees who were either pregnant or trying to get pregnant again at the time of enrolling in the writing course. Many of them felt ashamed or afraid to share the news about their new pregnancy, the failed artificial insemination, or miscarriages, because they did not want to hurt or revive intense emotions such as jealousy or despair among course participants, which was an actual risk as one participant shares in their journal: “*I am a petty person (a course participant in a similar situation as me has just told me she is pregnant, and I felt extra lonely and became grumpy with everything and everyone).*” In other words, the diversity of the life trajectories of the participants can create resistance, evoke unwanted emotions, and this is a challenge of peer support while each life trajectory is unique. This suggests that there are risks and limitations in peer sharing. At the same time, the exposure to this could potentially give participants experience with the everyday challenges of encountering children, parents and pregnant women in other contexts. Throughout email correspondence with the course facilitator and main researcher, some study participants shared the joy of being pregnant once again or having given birth to a new child. They also expressed that writing has helped them through the challenging emotions and anxieties of having a new child. Of course, given their age, health condition or civil status, not all participants have a chance to have more children. Therefore, an emphasis on development and life course could expand the understanding and potential treatment of their grief symptomatology.

One participant said during the plenary of the last session of group B “*my suffering has changed through time.*” When she read back what she had written during the 8 weeks of the course, she realized that suffering had not vanished but had transformed itself. Through writing, she described not only having developed a closer bond to her child, but also a closer relationship with herself. Most other participants in the plenary agreed that they felt “seen” and reflected in her words. Validating their suffering as an unavoidable experience, a part of our human condition, appeared to them as motivational. In addition to the hope arising from better understanding of their emotional world, most of our study participants in both online courses referred to a further insight: the more in contact with their suffering they became the more they felt motivated to serve others who have been through similar challenges. For instance, some of them shared their motivation to continue supporting each other as well as supporting others via volunteer activities, giving public speeches in hospitals or other venues, and making their writings available to the public. Turning unavoidable suffering into acts of service has been widely discussed as an attitude change that helps survivors of tragedies to find a sense of meaning in their lives (Frankl, 1969/2014). Therefore, in the context of this low-threshold intervention, psychoeducation about emotions, and the validation of the emotions that arose in class was crucial, even if it might not replace the need for psychotherapy to process these emotions further.

## Discussion

As described and analyzed above, most of the therapeutic writing practices involved a dialogue with different parts or selves of the participants, with their children, or with others. This was an intentional focus given the epistemological background of the primary authors, influenced by dialogical, narrative, and meanind-focused therapies (Hermans, 2001; Neimeyer, 2011; Lehmann, 2018a; Lehmann and Brinkmann, 2019c; Lengelle, 2021). Such a dialogical frame for self-exploration helped the participants focus on their identity before, during, and beyond the grief. For instance, given that all the study participants were women, their sense of womanhood emerged as an important theme in the written or oral reflections across the 8-week online course, inviting them to think of themselves as women, and not just as bereaved mothers. Viewed through a developmental lens, when adjusting to motherhood, women integrate not only the trajectories of what happens to them (i.e., having a child), but also shadow trajectories: all the other scenarios of what did not occur instead (i.e., having a steady income in a job in which they thrive) (Bastos, 2017). As we analyzed in the theme “a pregnancy that does not end,” the difference in this case is that the shadow trajectory becomes motherhood itself. Given their strong ambivalence regarding whether they have become and can still be considered as mothers compels a consideration of identity as a core psychological construct in understanding this specific kind of grief. This ambivalence appeared to shape their identities according where they were at in their life course. Some of our study participants had children from before. For some more, it was possible to think of a new pregnancy in the future and some participants were indeed pregnant by the time of enrolling the course. For others, the impossibility of a further pregnancy added a layer of complexity to their experience of grief. It would be then expected that the diversity of life trajectories among participants would make the death of a child affect also differently their sense of identity. That is, that the impact of the shadow trajectory of grief would vary depending of their individual contexts.

**FIGURE 1:**
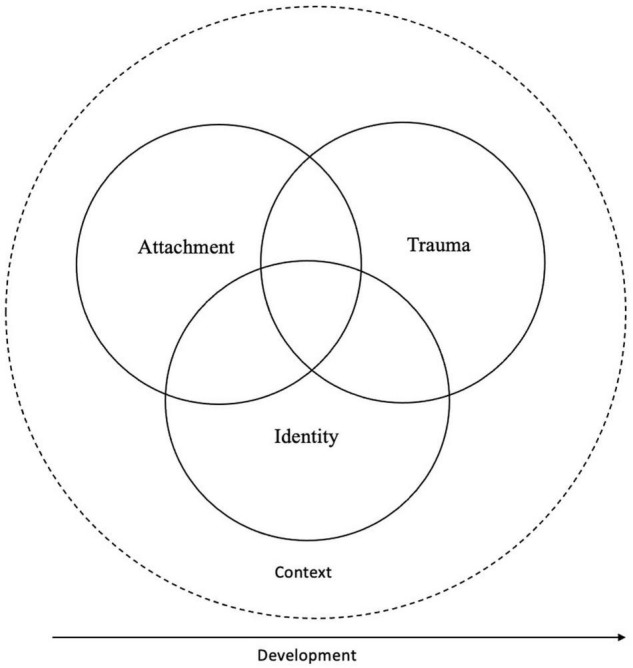
Integration of identity, trauma and attachment in grief theories.

Even if several empirical studies of grief and bereavement describe the impact that grief has on the sense of identity of those grieving (Neimeyer et al., 2006; Neimeyer, 2011), more theorization about identity in grief therapy is needed (Toller, 2008; Gillies et al., 2013). In contrast, most of the current theories of grief therapy relate to trauma or attachment approaches (Kosminsky and Jordan, 2016; Neimeyer, 2016) though more explicit reflections on grief and identity change is emerging in recent literature (Lengelle, 2021). In Figure 1, we include a developmental focus that can inform current trauma and attachment informed theories of grief, as well as an identity axis that could require more explicit theorization on its own. We also highlight the importance of situating the integration of these theoretical insights in a situational, relational and cultural context.

As part of the process of meaning reconstruction, many bereaved people perceive their sense of identity to change after experiencing the death of a child, as they integrate their grief in their everyday lives (Neimeyer et al., 2006). However, more research is needed when it comes to the relationship between identity development as a process of meaning making and coping responses to death-related losses (Gillies et al., 2013). Finding ways to continue and strengthen the bond with their child through rituals can be a way to promote the sense of identity as parents that many bereaved parents struggle with but desire to maintain (Toller, 2008). However, other research findings suggest that bereaved parents who develop symptoms of complicated grief have fewer alternative meaningful activities in their everyday lives (Bellet et al., 2020) and maintaining bonds with the deceased is not always healthy on its own (Bonanno, 2009). As a course facilitator, the first author of this article noted that those study participants who seemed to struggle the most with their grief were also those participants whose life’s meaning appeared to be centered solely around motherhood. In contrast, those participants who had other children, had a partnership, a job, or a hobby, manifested more anchors of meaning. This might support the idea that, in the context of grief, identity needs to be studied through dialogical lenses, putting more emphasis on the multivoicedness of the self (Toller, 2008). Some of the I-positions that participants expressed appeared more fluid and flexible than others. Promoting such multivoicedness of the self might be crucial for community or clinical interventions to be effective in restoring new life projects after the loss. This perspective is in line with meaning-centered grief therapy, which highlights the importance of addressing meaning, purpose, and identity as part of the therapeutic process for the integration of grief (Lichtenthal et al., 2019). However, this framework has more explicitly focused on reinforcing the sense of parental identity or in realigning one’s general sense of identity (Lichtenthal et al., 2019, 2022).

To our knowledge, dialogical and existential theories of identity could be integrated more successfully to inform the practice of grief therapies, as our findings suggest the possible benefit of combining them. Making sense of the death of a loved one, finding benefits from the grief, and identity development are at the core of therapeutic change because they enable the bereaved person to reprioritize life, focus on personal growth, and get a new sense of the future, among other outcomes (Gillies and Neimeyer, 2006). As our study participants experienced it, the metaphor of their mind being a theater with different characters enabled them to honor their identity as a process, rather than a state. Acknowledging their multivoicedness helped them to also contemplate grief as a human experience, in addition to considering its impact on their sense of womanhood, motherhood, or partnership, just to name a few positions. That is, given that grief is a boundary experience that represents a sense of rupture in the actual or imagined flow of our everyday lives (Yalom, 1980; Zittoun, 2006), the integration of grief could be also understood to include self-exploration and identity transformation as a central process, instead of being portrayed as an incidental process in more trauma or attachment-oriented models. Instead, each of these three dimensions of identity, trauma and attachment can be considered as an equally important and interdependent dimension in meaning-focused grief theory (Neimeyer, 2022). As we have emphasized in the previous subsections of this article, self-exploration or, in words of the participants themselves, a better understanding of who they are and who they want to become, were some of the most enriching learnings of the online course. One of the reasons why the participants experienced such a process of self-exploration as effective in writing courses could be due to the sense of reciprocity and support from the group, as reported in other qualitative studies of writing courses (Lehmann and Brinkmann, 2019a).

### Limitations

Longer term writing courses might also be considered, to foster deeper meaning-making and emotional processing, as well as the reconstruction of participant identities and life projects despite and beyond the grief; 8 weeks may be too brief given the profundity of tragic bereavement. For instance, some participants mentioned they would have preferred to meet 1, 5 or 2 h per week, over a longer period of time. Different combinations of length of the course might be considered in terms of feasibility for the facilitator(s). Low-threshold interventions such as the present online writing course could be particularly insufficient for some mothers who have experienced other trauma in their lives, as well as for those who have disorganized, anxious or avoidant attachment styles. Tools for assessing trauma and attachment can be included in further screenings for determining the suitability of the courses. In the same line of ideas, a further differentiation of the experience of grief by type of death (e.g., stillbirth and illness) could shed light to more specific needs of bereaved parents. Other interventions such as psychotherapy and further peer support are important, especially for those mothers who continue to need support in their ongoing grief following the course. Given the current lack of emotional support offered to bereaved parents in Norway, both the development of low-threshold community interventions and professional clinical interventions is needed.

### Implications for Research

The present study indicates several paths for future research. For example, having more access to the contents of the journals of study participants could benefit the study of the processual nature of meaning-making in grief. As the summary table that one of our participants contributed to us illustrates, the fluctuation of emotion in association with self-criticism, group dynamics, and the course of grief itself could shed light on important nuances of the integration of attachment, trauma, and identity theories for the treatment of grief and bereavement. In addition, longer follow-ups via in-depth interviews could expand the insights we gained from the data we collected via fieldwork, journaling, and surveys. Further studies could, for instance, follow up on the diverse trajectories of grief of bereaved parents, considering individual differences such as whether they have other living children, are pregnant at the time of the intervention, are attempting to conceive further children via artificial insemination or are pursuing adoption. Other studies, if integrating practices of therapeutic writing into formal clinical interventions, could also ran randomized control trials in order to either compare the effectiveness of therapeutic writing in contrast with other interventions, or compare the effectiveness of different techniques in therapeutic writing.

### Implications for Clinical and Community Practices

The findings in this study have several implications for clinical and community practices. Given that bereaved parents have different emotional needs and are met at different phases of both their grief and of their life trajectories, it is crucial for them to have access to diverse support offers through time. Perhaps one of the greatest insights around grief is not about how it is resolved or healed, given that grief is an inherent part of the human condition. It is about not being alone in grief, finding communities of support that can compensate the lack or the limits of existing professional or cultural support systems for grief, bereavement and mourning.

As we have illustrated in this article, therapeutic writing is a powerful tool to promote self-exploration, enhance emotional literacy, and foster insight into the nature of grief. Specific writing tasks can be used both in one-to-one or group interventions, with the compassionate and attentive follow-up of facilitators who have personal and or professional competence around grief as well as writing itself. In addition, once bereaved parents have learnt these techniques and have knowledge about the course of grief, therapeutic writing could be potentially used as a self-help method. A further outcome of this project and as supported by a further grant that the first author received from the DAM Foundation, a workbook will be developed and made freely available for LUB members to support this goal.

### Conclusion

Throughout this article we have explored the ways in which bereaved mothers experienced attending an 8-week therapeutic writing online course to support the integration of their grief. The meaning units that we crafted to portray the experiences of the participants were: (1) Where does my story begin? the “both and” of their silent chaos; (2) Standing on the middle line: a pregnancy that does not end; (3) Closures and openings: “careful optimism” and the need for community support. After conducting a existential-phenomenological analysis of the data, we conclude that a course on therapeutic writing is not just a writing course, it is a platform to promote the belonging and community that bereaved parents need to process difficult emotions and find meaning beyond the trauma of having experienced the death of a child. This online platform holds promise as a low-threshold intervention. The reciprocity and sense of belonging that our participants experienced became as crucial for them as the writing practices themselves. This sense of belonging is considered a deep existential need which holds a space for grief, not only as a phenomenon which symptoms are to be treated, but as a human experience which is to be attended with compassion.

This study also sheds light on the depth and intensity of the emotional processes that bereaved mothers go through as they attempt to integrate grief in their everyday lives after the unexpected death of a child. Such grief involves not only sadness but also anger, guilt, shame and fear which need to be differentiated and processed. While writing is a tool that supports such differentiation of emotional categories as well as their processing (i.e., allowing those grieving to find the words they are yearning for), it is important that bereaved parents also receive support beyond peer groups or low-threshold interventions such as ours. Because mourning the death of a child is rarely a brief and simple process, multiple resources such as family support, psychotherapy, peer groups, and community interventions such as therapeutic writing courses can complement and enhance one another.

Given that facing the death of a loved one or embracing our own dying processes involves the paradox of trying to make sense of that which doesn’t, writing can be a tool for self-exploration, consolation, and creativity that can support meaning-making of the experience of grief, mourning and bereavement. The proposed therapeutic writing intervention is distinctive from previous approaches, as it: (a) explicitly focuses on emotional processing; (b) uses aesthetic and poetic resources to support legacies and continuing bonds with the lost children; and (c) promotes a sense of reciprocity and community among the participants of the course, given that their sharing of experiences can not only be motivational for one another, but also support their mutual post-traumatic growth; (d) prioritizes relational and self-exploratory practices over trauma exposure, especially because it is designed as a low threshold intervention and not a clinical treatment. That is, this writing course is not just about the act of writing as a therapeutic tool, it is aimed at promoting a community of belonging and peer-support that can be self-sustained by the attendees after the intervention ends.

In addition to the exploration of their emotional world, exploration of participants’ identities was welcomed in helping them make sense of the death of their children. As we have suggested, an integration of dialogical and existential approaches to identity was experienced as beneficial by the participants: they developed a deeper sense of their human condition as persons, women, mothers, workers, and other I-positions that represented aspects of their inner and outer world. This multivoiced perspective consequently enabled them to also mind their inner critics and feel more compassion for themselves and others. However, a focus on identity in itself does not suffice: it is crucial to view bereavement through developmental and contextual lenses to better understand participants’ grief, and why some of their grief symptoms are prolonged through time. In connection with this, the validation of their suffering, and the quest for meaning throughout their suffering motivated them to engage not only in the various writing practices, but also to share these writings, and to participate actively in the subgroup plenary reflections throughout the online course.

## Data Availability Statement

The datasets cannot be available to the public because of our aim to ensure the protection of the participants’ confidentiality around sensitive information. In the “Materials and Methods and Results” sections, as well as in the supplementary material, we have provided excerpts of the data in anonymized form and (or) with the approval of specific participants. Requests to access the datasets should be directed to OL.

## Ethics Statement

The studies involving human participants were reviewed and approved by all requirements of the ethical regulations for scientific research in Norway were fulfilled (NSD approval number 176014 and granted exemption from REK). In addition, and as a preventive measure, we included up to 2 sessions of emotional counseling for each of the participants with the above-mentioned group of specially trained emotion-focused therapists. The patients/participants provided their written informed consent to participate in this study. Written informed consent was obtained from the individual(s) for the publication of any potentially identifiable images or data included in this article.

## Author Contributions

OL designed the intervention, facilitating the online courses, collecting and analyzing the data, as well as writing the drafts of the article and integrating feedback from co-authors into the final version of the manuscript. RN and RL consulted on the design of the intervention, provided mentoring during the intervention process, and offered detailed feedback on the manuscript. JT contributed by co-designing the methodology, as well as providing input on the analysis of the data, and the theoretical development of the introduction and discussion of the article. AH contributed by supporting the structure of the methodology, results, limitations, and implications sections of the article. TK contributed to the development of the grant application, as well as the recruiting of the study participants, the ethical considerations and submission of the project to the research committees in Norway. She also contributed to the development of the discussion of the article. All authors contributed to the article and approved the submitted version.

## Conflict of Interest

The authors declare that the research was conducted in the absence of any commercial or financial relationships that could be construed as a potential conflict of interest.

## Publisher’s Note

All claims expressed in this article are solely those of the authors and do not necessarily represent those of their affiliated organizations, or those of the publisher, the editors and the reviewers. Any product that may be evaluated in this article, or claim that may be made by its manufacturer, is not guaranteed or endorsed by the publisher.
